# Radiomics combined with clinical characteristics predicted the progression-free survival time in first-line targeted therapy for advanced non-small cell lung cancer with EGFR mutation

**DOI:** 10.1186/s13104-022-06019-x

**Published:** 2022-04-14

**Authors:** Jian-man Zhu, Lei Sun, Linjing Wang, Tong-Chong Zhou, Yawei Yuan, Xin Zhen, Zhi-Wei Liao

**Affiliations:** 1 Department of Radiation Oncology, Affiliated Cancer Hospital & Institute of Guangzhou Medical University, Guangzhou, 510095 Guangdong China; 2 Department of Radiation Oncology, Affiliated Zhujiang Hospital of Southern Medical University, Guangzhou, 510280 Guangdong China; 3 Radiotherapy Center, Affiliated Cancer Hospital & Institute of Guangzhou Medical University, Guangzhou, 510095 Guangdong China; 4 School of Biomedical Engineering, Southern Medical University, Guangzhou, 510515 Guangdong China

**Keywords:** Non-small cell lung cancer, EGFR-TKI, Radiomics, Machine learning

## Abstract

**Objective:**

This study was to explore the most appropriate radiomics modeling method to predict the progression-free survival of EGFR-TKI treatment in advanced non-small cell lung cancer with EGFR mutations. Different machine learning methods may vary considerably and the selection of a proper model is essential for accurate treatment outcome prediction. Our study were established 176 discrimination models constructed with 22 feature selection methods and 8 classifiers. The predictive performance of each model were evaluated using the AUC, ACC, sensitivity and specificity, where the optimal model was identified.

**Results:**

There were totally 107 radiomics features and 7 clinical features obtained from each patient. After feature selection, the top-ten most relevant features were fed to train 176 models. Significant performance variations were observed in the established models, with the best performance achieved by the logistic regression model using gini-index feature selection (AUC = 0.797, ACC = 0.722, sensitivity = 0.758, specificity = 0.693). The median R-score was 0.518 (IQR, 0.023–0.987), and the patients were divided into high-risk and low-risk groups based on this cut-off value. The KM survival curves of the two groups demonstrated evident stratification results (*p* = 0.000).

**Supplementary Information:**

The online version contains supplementary material available at 10.1186/s13104-022-06019-x.

## Introduction

The EGFR-TKIs (epidermal growth factor receptor-Tyrosine kinase inhibitors) such as gefitinib and erlotinib are used as the first-line treatment for NSCLC (non-small cell lung cancer) patients with EGFR mutation. However, acquired resistance is usually observed after 9–13 months of evident tumor response [1]. Moreover, primary resistance would tend to affect the therapeutic efficacy of TKIs by leaving with only a few months of reaction time. In this sense, exploring an effective prognostic marker to predict the development of TKIs resistance of EGFR positive patients will be clinically meaningful to allow physicians to timely adjust the treatment strategies.

Radiomics is able to extract quantitative imaging biomarker regarding the biological, prognostic and predictive hidden information from medical images. Successful application of CT-based (computed tomography) radiomics has witnessed in NSCLC prediction and prognosis [2], in particular for prediction of EGFR mutations [3, 4], and EGFR-TKI prognosis [5, 6]. These studies empirically adopted different ML (machine learning) methods to build the predictive model, e.g., RF (random forest) [3, 4] or LASSO regression [5–7], it is still unclear how to choose an appropriate ML algorithm for modeling and which model is more superior over the other.

The goal of current study is to explore the most appropriate radiomics modeling method to predict the progression-free survival of EGFR-TKI treatment in advanced non-small cell lung cancer with EGFR mutations. In order to screen patients suitable for EGFR- TKI therapy.

## Main text

### Patients

A total of 100 patients with stage IIIB-IV EGFR positive NSCLC patients treated with EGFR-TKIs in the Affiliated Cancer Hospital of Guangzhou Medical University between January 2016 and December 2019 met the inclusion criteria and were finally recruited. The inclusion criteria were as follows: (1) stage IIIB-IV lung adenocarcinoma confirmed by histopathology; (2) patients of EGFR mutations with confirmed 19del or 21L858R; (3) no treatments received prior to EGFR TKIs therapy; (4) the EGFR-TKIs therapy was used as the first-line treatment; (5) with complete CT images within 2 weeks before EGFR-TKIs treatment. The exclusion criteria included: (1) age less than 18 years old; (2) underwent any other antitumor therapies; and (3) with incomplete clinical records or CT images.

Patients with 19del or 21L858R mutations have received gefitinib, erlotinib, or other EGFR-TKIs as first-line treatment. Drugs were orally administrated daily until disease progressed. Treatment efficacy evaluations included routine laboratory tests and chest CT scans at least every 4–12 weeks. PFS was estimated from the beginning time of EGFR-TKIs therapy to the date of disease progression or death. The outcome is assessed within 3 months of the targeted therapy via the RECIST1.1 criterion. Patients’ clinical characteristics including sex, age, stage, smoking status, mutations, TKIs and outcome are summarized in Additional file 1: Table S1.

### Methods

#### Image acquisition and feature extraction

Pretreatment CT scans were acquired after intravenous injection of 100 ml ioversol (Heng Rui Pharmaceuticals, Jiangsu China), with scanning parameters of 120 kV, 160 mAs, 0.6 s rotation time, and image matrix size of 512 × 512. All patient images were stored in DICOM format. The VOI (volume of interest) on CT images was delineated independently by two radiologists using 3D-slicer software (slicer4.10.2, https://www.slicer.org). The VOI delineation was performed slice-by-slice on the CT images with standard mediastinal (window width, 400 HU; window level, 40 HU) and lung (window width, 1500 HU; window level, − 700 HU) window settings. The conformity of delineated VOIs were measured by the Dice similarity coefficient. The two delineated VOIs with Dice index greater than 0.9 were averaged to yield the final VOI. Discrepancies on the lesion boundary (Dice < 0.9) were resolved by further discussions until mutual consensus were reached.

Radiomics features extraction were conducted within the VOIs utilized an open-source python package Pyradiomics [8]. Extracted features (*n* = 107, Additional file 1: Table S2) included: (1) first order features (*n* = 18); (2) shape features (*n* = 14); (3) GLCM (gray level co-occurrence matrix) features (*n* = 24); (4) GLSZM (gray level size zone matrix) features (*n* = 16); (5) GLRLM (gray level run length matrix) features (*n* = 16); (6) NGTDM (neighboring gray tone difference matrix) features (*n* = 5); (7) GLDM (gray level dependence matrix) features (*n* = 14).

#### Prediction modeling

Noted that our goal was to identify the most significant variables that could discriminate patients with fast and slow progression. Thus, the dimension reduction was necessary to improve the accuracy in the later step of building the machine learning model for classification. Therefore, the feature selection was conducted before constructing the models, and the most related features to this study were selected.

Prediction modeling was performed on 107 radiomics features combined with 7 clinical features (including sex, age, stage, smoking status, mutations, TKI, and outcome). A specific model was constructed by a feature selection procedure followed by a particular classifier. In this study, 22 feature selection methods and 8 classifiers (Additional file 1: Table S3) were studied, and therefore resulting in 176 prediction models (different combinations of ‘feature selection’ + ‘classifier’) to be evaluated. In each prediction model, we empirically set the number of selected features to 10 to balance the patient sample size vs. feature numbers.

The prediction model was evaluated via a repeated (5 times) five-fold CV (cross-validation), where 80% and 20% of the dataset were respectively reserved for model training and validation. To reduce the effect of the imbalance, the SMOTE (synthetic minority oversampling) [9] technique was applied on the training set in each fold of the CV. The prediction performances were quantified by the area under the AUC (receiver operating characteristic (ROC) curve), ACC (accuracy), SEN (sensitivity), and SPE (specificity).

#### Statistical analysis

All statistical analyses were conducted using the SPSS (version 22.0). Comparison between groups of classified variables used chi-square test. The Kaplan–Meier algorithm was used to estimate the survival curves that were compared by the log-rank test. Normality of data distribution was assessed by the Kolmogorov–Smirnov test. The student’s t-test was used for normally distributed continuous variables and the Mann–Whitney U test was used for non-normally distributed continuous variables. A *p*-value of < 0.05 was regarded as statistically significant.

### Results

#### Patient characteristics

A median PFS (10 months) of the whole patient cohort was used to divide patients into a rapidly progressing group (*n* = 49) and a slowly progressing group (*n* = 51). The median age of all patients was 59 years. More patients with 19del mutation were found in the slow-progression group (19del vs 21L858R, 59.6% vs 37.5%); In contrast, more 21L858R mutation patients were found in the fast-progression group (19del vs 21L858R, 40.4% vs 62.5%). Significant difference in mutation site of the two groups was observed (*p* = 0.03). In the slow progression group, 44 (63.8%) patients exhibited ‘CR (complete remission)’ or ‘PR (partial remissions)’ after EGFR-TKIs therapy, while only 5 (16.1%) patients were categorized as ‘SD (stable diseases)’ or ‘PD (progressive diseases)’ (by RECIST1.1 criteria). In contrast, in the rapid-progression group, only 25 patients (36.2%) achieved CR or PR after EGFR-TKIs therapy, and 26 patients (83.9%) were classified as SD or PD. The clinical factor of ‘CR + PR’ outcome was found to be associated with better prognosis (*p* = 0.03) by the univariate analysis.

#### Model performance comparisons

The 107 radiomics features and 7 clinical features were obtained from each patient. In each of the 176 model, the top-ten most relevant features were selected by feature selection and fed to train a classifier in each model. The performances of the 176 models are depicted as a AUC heatmap as shown in Additional file 1: Fig. S1. The other quantitative performance metrics as such ACC, SEN, SPE of each model were detailed in Additional file 1: Fig. S2. Evident prediction performance variations were observed in the evaluated models. The average AUC of all 176 models is 0.591 with the maximum AUC of 0.797 achieved by the model built with LR (Logistic Regression) and gini-index feature selection (ACC = 0.722, SEN = 0.758, SPE = 0.693). While the worst model (SVM (support vector machine) and JMI (joint mutual information) feature selection) only yield AUC = 0.371, ACC = 0.559, SEN = 0.611 and SPE = 0.507. The performance of the best 10 models was listed in Table 1. The mean AUC tends to significantly (*p* = 0.000) decline to 0.524 if only radiomics features were used for modeling, as compared with the AUC heatmap shown in Additional file 1: Fig. S3. Each of the 176 models were respectively trained by all the features (*n* = 114, including 107 radiomics and 7 clinical features) and cross-validated by the five-fold cross-validation. The most relevant features had been selected in each model, and we can count and rank their frequencies of being selected as the top feature, as shown in Table 2. The top-ten features most selected were marked in blue in Additional file 1: Fig. S4. Among these top-ten features, four features including two texture features (glcm-difference variance: 14.59 ± 6.63 vs. 18.16 ± 8.71, *p* = 0.024; glszm-small area emphasis: 0.68 ± 0.04 vs. 0.70 ± 0.03, *p* = 0.003) and two clinical features (mutation, *p* = 0.03; outcome, *p* = 0.000) showed a statistically significant difference between the slow progression group and the fast progression group.

**Table 1:** Feature selection methods and classifiers for the top ten models

Classifier	Feature selection method	AUC	ACC	Sensitivity	Specificity
Logistic	gini-index	0.797	0.722	0.758	0.693
Logistic	ll-l21	0.763	0.681	0.716	0.653
Bagging	CIFE	0.764	0.671	0.636	0.707
Logistic	reliefF	0.759	0.662	0.676	0.655
Bagging	MRMR	0.743	0.662	0.616	0.729
Bagging	MIFS	0.742	0.670	0.656	0.673
Adaboosting	f-score	0.740	0.671	0.756	0.593
SVM	MRMR	0.739	0.712	0.733	0.695
SVM	CIFE	0.734	0.692	0.713	0.676
SVM	MIFS	0.734	0.712	0.733	0.695

**Table 2:** The top ten features and the corresponding mean (± SD) value (or median (IQR)) and the *p*-value between the slow and fast progression groups

Feature category		Feature	Slow-progress	Fast-progress	*p*-value
Shape-based (n = 4)		Elongation	0.76 ± 0.12	0.71 ± 0.13	0.067^a^
		Least Axis Length	30.06 ± 11.91	20.53 ± 8.85	0.234^a^
		Flatness	0.59 ± 0.14	0.55 ± 0.13	0.229^a^
		Major Axis Length	52.25 (19.52,110.61)	46.56 (23.28,145.60)	0.858^b^
First-order based (n = 1)		Inter quartile range	140 (36,560)	154 (42,385)	0.962^b^
Texture	GLSZM (n = 1)	Small Area Emphasis	0.68 ± 0.04	0.70 ± 0.03	0.003^a^
	GLCM (n = 1)	Difference variance	14.59 ± 6.63	18.16 ± 8.71	0.024^a^
Clinical based (n = 3)		Smoke	–	–	0.238^c^
		Mutation	–	–	0.030^c^
		Outcome	–	–	0.000^c^

^a^t-test

^b^Mann–Whitney U test

^c^Chi-square test

#### Prognostic performance

**Fig. 1:**
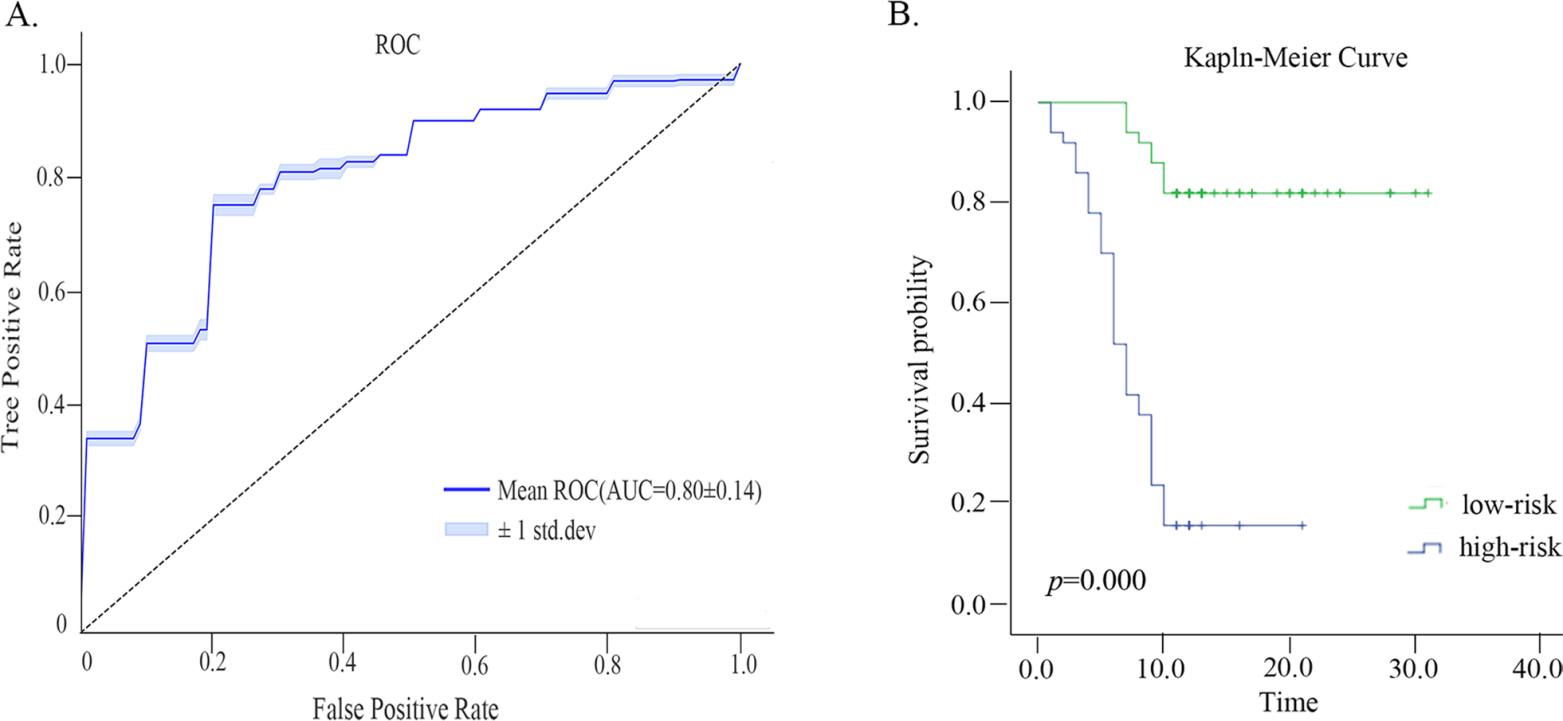
**A** Time-dependent ROC curves of the “gini-index-Logistic regression” model of using the top-10 features at 10 months. **B** Kaplan Meier survival curves of EGFR positive NSCLC patients. The *p*-values were calculated using the log-rank tests

We stratified patients by the R-score (risk score) given by the best model, i.e., the Gini-index-LR model evaluated from the 176 models. The estimated median R-score 0.518 (IQR, 0.023–0.987) was used as the cut-off value to stratify the high-risk group from the low-risk group. The higher the R value, the higher the likelihood of rapid progression. The KM (Kaplan–meier) method and the Log-Rank test were used to evaluate and compare the survival curves of the high-risk group and the low-risk group. The ROC and KM survival curves (*p* = 0.000) of Gini-index-LR method were shown in Fig. 1.

### Discussion

A particular prediction model can be built with a feature selection followed by a classifier. Given a large pool of available feature selection methods and different classifier algorithm, their combinations may result in a large number of prediction models to choose from. In this study, we constructed and comprehensively evaluated 176 models using 22 feature selection methods and 8 classifiers. The best model method was gini-index-logistic (AUC = 0.797). The R-score derived from the gini-index-logistic model was validated on the KM curve, and had also achieved satisfactory stratification result. We have observed that the other models, e.g., the CIFE-Bagging (AUC = 0.764), the ll-l21-LR (AUC = 0.763), and the ReliefF-LR (AUC = 0.759) also demonstrated high predictive performances. In general, the predictive models built with the LR classifier seemed to perform better than those models built with the RF classifier, which has been used in previous investigations for EGFR mutations prediction modeling [4].

In this study, we have identified two CT image textural features, i.e., the GLCM-derived feature "DV (Difference Variance)" and the GLSZM-based feature "SAE (Small Area Emphasis)" to be significantly associated with progression (*p* = 0.003 and *p* = 0.024). The DV is a measure of heterogeneity that places higher weights on differing intensity level pairs that deviate more from the mean. The lesions on the fast-progression group (DV: 18.16 ± 8.71) seemed to be more heterogeneous than the slow-progression group (DV: 14.59 ± 6.63). While the SAE is a measure of the distribution of small size zones [8]. The fast-progression group had greater SAE values than the slow-progression group (0.70 ± 0.03 vs. 0.68 ± 0.04), which was indicative of more smaller size zones and more fine textures from the lesion on CT image that might be correlated with progression.

Similar to Hong et al. [10] study, we also found that the EGFR mutation type was indicative of patient prognosis. In addition, we identified complete or partial remission (CR or PR) within 3 months after EGFR treatment as a prognostic factor. This finding was consistent with clinical observations and previous studies, e.g., Mizuki et al. [7] claimed that the proportional volume change at 8 weeks was related with overall survival in EGFR-mutant advanced NSCLC patients treated with first-line EGFR-TKIs.

In summary, our study screened out an optimal model to predict progression-free survival time of NSCLC patients treated with first-line EGFR-TKI within a machine learning based framework.

### Limitations

This study has several limitations need to be addressed. First, this was a single institutional study where the patient sample size was relatively small. An independent external validation cohort was lacked to confirm the generalization capability of the model and the associated findings presented here. Second, we didn't delve into the effect of the number of features on the model. Third, the number of the clinical factors studied in the model were relatively small. We have observed increased AUC values when clinical features were incorporated into the model rather than using image texture feature alone. Improved model performance might be expected if more clinical or pathological factors were embedded.

## Supplementary Information

**Additional file 1: Table S1** Demographics and characteristics of the 100 patients. **Table S2** Radiomics features. **Table S3** Feature selection methods and classifiers used for discrimination models. **Figure S1** Heatmap representing AUC of 176 models was constructed from the clinical features and radiomics features. **Figure S2** Heatmap representing the ACC(A),SEN(B) and SPE(C) of 176 models constructed from clinical features and radiomic features. **Figure S3** Heatmap representing AUC of 176 models constructed from radiomic features. **Figure S4** Histogram of the number of times that each feature is selected in the five-fold cross validation.

## Data Availability

The data that support the findings of our study are available from the Affiliated Cancer Hospital of Guangzhou Medical University but restrictions apply to the availability of these data, which were used under license for the current study, and so are not publicly available. Data are however available from the authors upon reasonable request and with permission of the Affiliated Cancer Hospital of Guangzhou Medical University.

## Abbreviations

EGFR-TKIs: Epidermal growth factor receptor-Tyrosine kinase inhibitors
NSCLC: Non-small cell lung cancer
PFS: Progression-free survival
CT: Computed tomography
ML: Machine learning
RF: Random forest
VOI: Volume of interest
GLCM: Gray level co-occurrence matrix
GLSZM: Gray level size zone matrix
GLRLM: Gray level run length matrix
NGTDM: Neighboring gray tone difference matrix
GLDM: Gray level dependence matrix
CV: Cross-validation
SMOTE: Synthetic minority oversampling
AUC: Receiver operating characteristic (ROC) curve
ACC: Accuracy
SEN: Sensitivity
SPE: Specificity
CR: Complete remission
PR: Partial remissions
SD: Stable diseases
PD: Progressive diseases
LR: Logistic Regression
SVM: Support vector machine
JMI: Joint mutual information
R-score: Risk score
KM: Kaplan–Meier
DV: Difference Variance
SAE: Small Area Emphasis
