# Supplementary material for: Radiomics combined with clinical characteristics predicted the progression-free survival time in first-line targeted therapy for advanced non-small cell lung cancer with EGFR mutation
Source: BMC Res Notes. 2022 Apr 14;15:140. doi: 10.1186/s13104-022-06019-x (PMC9008953; doi:10.1186/s13104-022-06019-x)
Supplement: Supplementary file 1 — Additional file 1: Table S1 Demographics and characteristics of the 100 patients. Table S2 Radiomics features. Table S3 Feature selection methods and classifiers used for discrimination models. Figure S1 Heatmap representing AUC of 176 models was constructed from the clinical features and radiomics features. Figure S2 Heatmap representing the ACC(A),SEN(B) and SPE(C) of 176 models constructed from clinical features and radiomic features. Figure S3 Heatmap representing AUC of 176 models constructed from radiomic features. Figure S4 Histogram of the number of times that each feature is selected in the five-fold cross validation. [file 13104_2022_6019_MOESM1_ESM.docx]

**Table S1.** Demographics and characteristics of the 100 patients. A median PFS of 10 months was used to divide the patient cohort into the fast-progression and slow-progression groups.

| Characteristic | total  n=100 |  | slow-progress set  n=49 |  | fast-progress set  n=51 | *p*-value |
| --- | --- | --- | --- | --- | --- | --- |
| age |  |  |  |  |  | 1.000 |
| ≤59 | 50(50.0%) |  | 25(50.0%) |  | 25(50.0%) |  |
| ＞59 | 50(50.0%) |  | 24(48.0%) |  | 26(52.0%) |  |
| sex |  |  |  |  |  | 0.212 |
| men | 36(36.0%) |  | 21(58.3%) |  | 15(41.7%) |  |
| women | 64(64.0%) |  | 28(43.8%) |  | 36(56.2%) |  |
| Smoking status |  |  |  |  |  | 0.238 |
| Smoker | 23(23.0%) |  | 14(60.9%) |  | 9(39.1%) |  |
| Nonsmoker | 77(77.0%) |  | 35(45.5%) |  | 42(54.5%) |  |
| Stage |  |  |  |  |  | 0.262 |
| IIIB+IIIC+IVA | 27(27.0%) |  | 16(59.3%) |  | 11(40.7%) |  |
| IVC | 73(73.0%) |  | 33(45.2%) |  | 40(54.8%) |  |
| Mutation |  |  |  |  |  | 0.030 |
| 19del | 52(52.0%) |  | 31(59.6%) |  | 21(40.4%) |  |
| 21L858R | 48(48.0%) |  | 18(37.5%) |  | 30(62.5%) |  |
| TKI |  |  |  |  |  | 0.633 |
| Gefitinib | 56(56.0%) |  | 29(51.8%) |  | 27(48.2%) |  |
| Erlotinib | 13(13.0%) |  | 7(53.8%) |  | 6(46.2%) |  |
| other | 31(31.0%) |  | 13(41.9%) |  | 18(58.1%) |  |
| Outcome |  |  |  |  |  | 0.000 |
| CR+PR | 69(69.0%) |  | 44(63.8%) |  | 25(36.2%) |  |
| SD+PD | 31(31.0%) |  | 5(16.1%) |  | 26(83.9%) |  |

**Table S2.** Radiomics features.

| Feature type | Numbers | Features |
| --- | --- | --- |
| Shape features | 14 | elongation,flatness,least axis length,major axis length,maximum 2D diameter column,maximum 2D diameter row,maximum 2D diameter slice,maximum 3D diameter,mesh volume,minor axis length,sphericity,surface area,surface volume ratio,voxel volume |
| First-order features | 18 | 10 percentile,90 percentile,energy,entropy, interquartile range,kurtosis,maximum,mean absolute deviation,mean,median,minimum,range,  robust mean absolute deviation,root mean squared,skewness,total energy,uniformity,variance |
| Texture features | 75 | GLCM(N=24),GLRLM(N=16),GLSZM(N=16),  GLDM(N=14),NGTDM(N=5) |

**Table S3** Feature selection methods and classifiers used for discrimination models.

| **Feature selection methods**^*1^**(*n*=22)** | CIFE^[1]^,CMIM^[1]^,DISR^[1]^,ICAP^[1,2]^,JMI^[1]^,MIFS^[1]^,MIM^[1]^,MRMR^[1]^,Fisher_score^[3]^,Lap_score^[3]^,ReliefF^[4]^,SPEC^[5]^,Trace_ratio^[6]^,II_I21^[7]^,Is_I21^[7]^,MCFS^[8]^,NDFS^[9]^,RFS^[10]^,UDFS^[11]^,F_score^[2]^,Gini_index^[2]^,T_score^[2]^ |
| --- | --- |
| **Classifiers**^*2^  **(*n*=8)** | Logistic regression,SVM,Naïve Bayes,KNN,Decision tree,Bagging, Random forest, AdaBoosting |
| *1. Implemented with an open source machine learning toolkit- scikit-feature^[2]^  *2. Implemented with an open source machine learning toolkit- scikit-learn^[12]^ | |

**Figure S1**

**
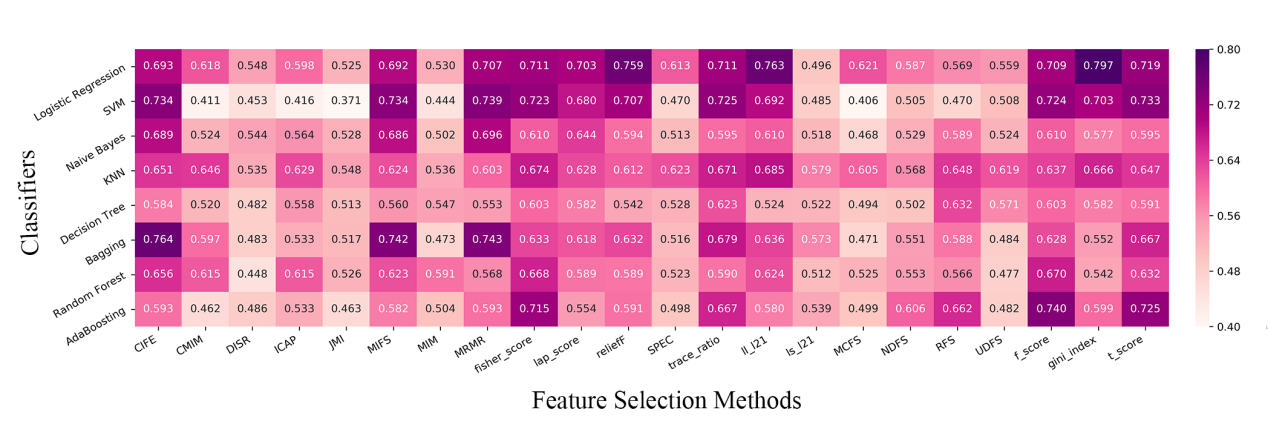
**

Figure S1.Heatmap representing the predictive performance (AUC) of multiple prediction models built with different feature selection methods (in rows) and different classification methods (in columns). all the 176 models used top 10 features inclued radiomic features and clinical features.

**Figure S2**

A


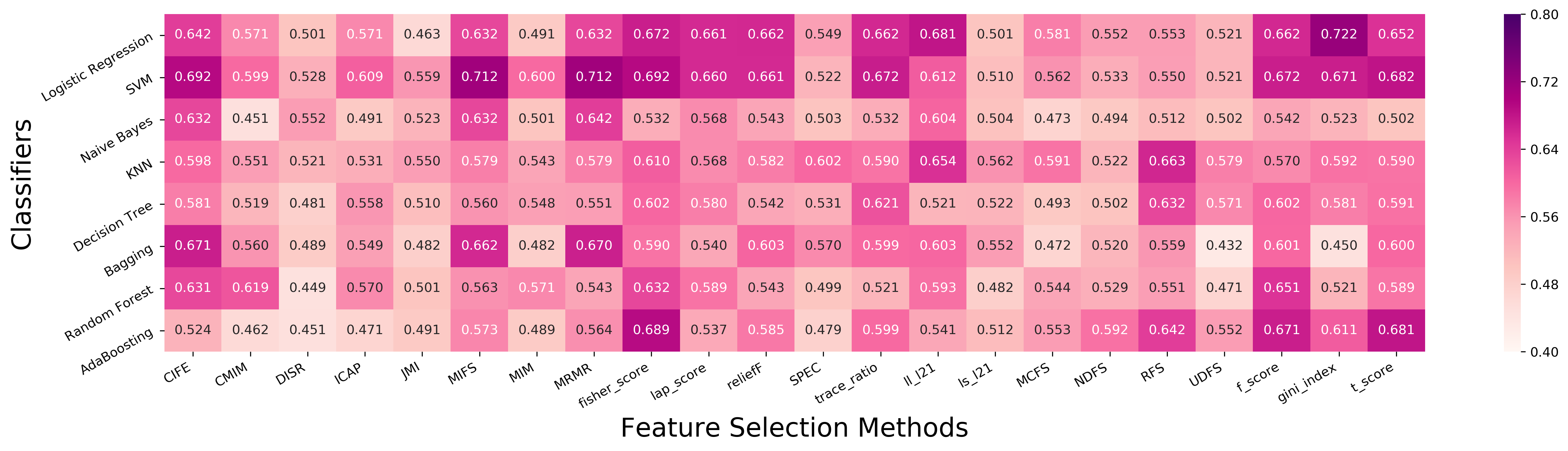


B


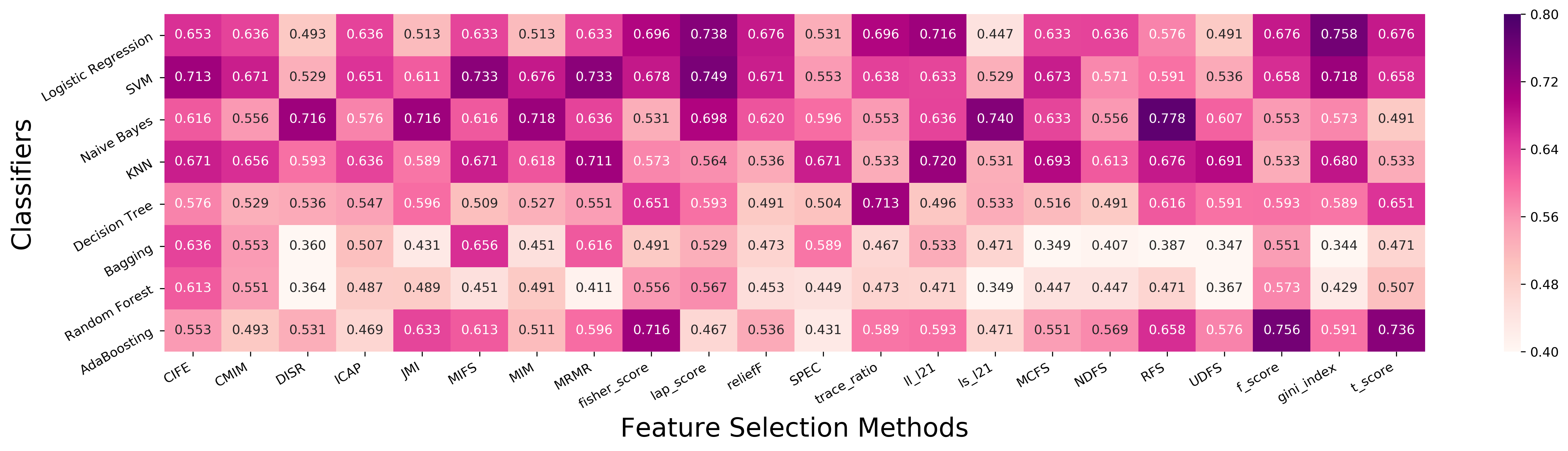


C


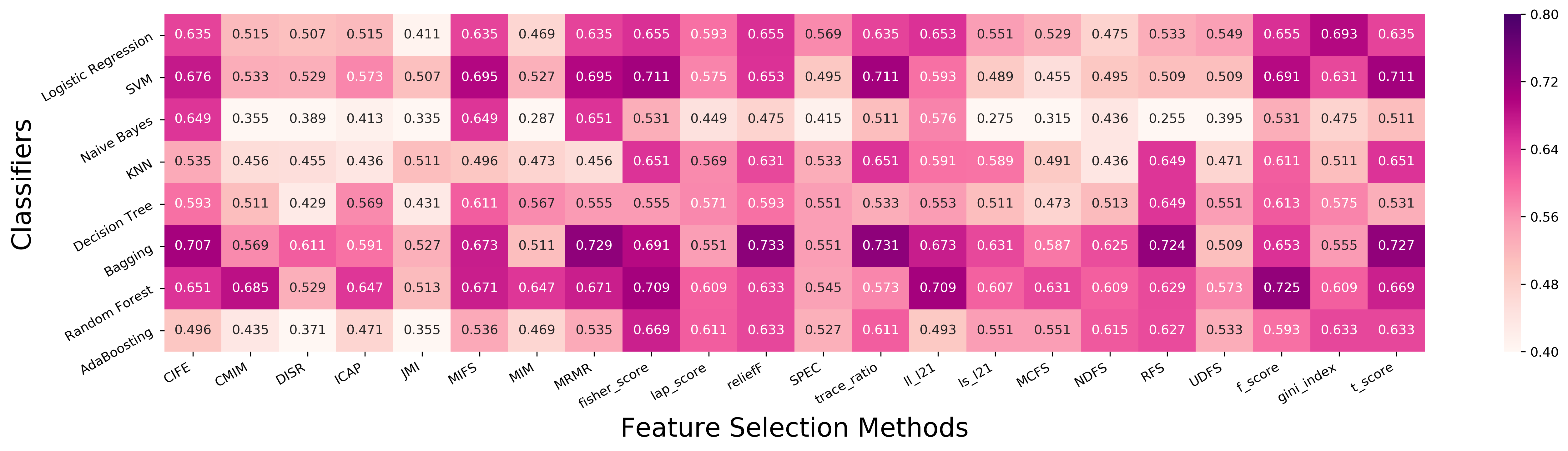


Figure S2. Heatmap representing the ACC(A),SEN(B) and SPE(C) of all the 176 discriminative models used top 10 features inclued radiomic features and clinical features.

**Figure S3**
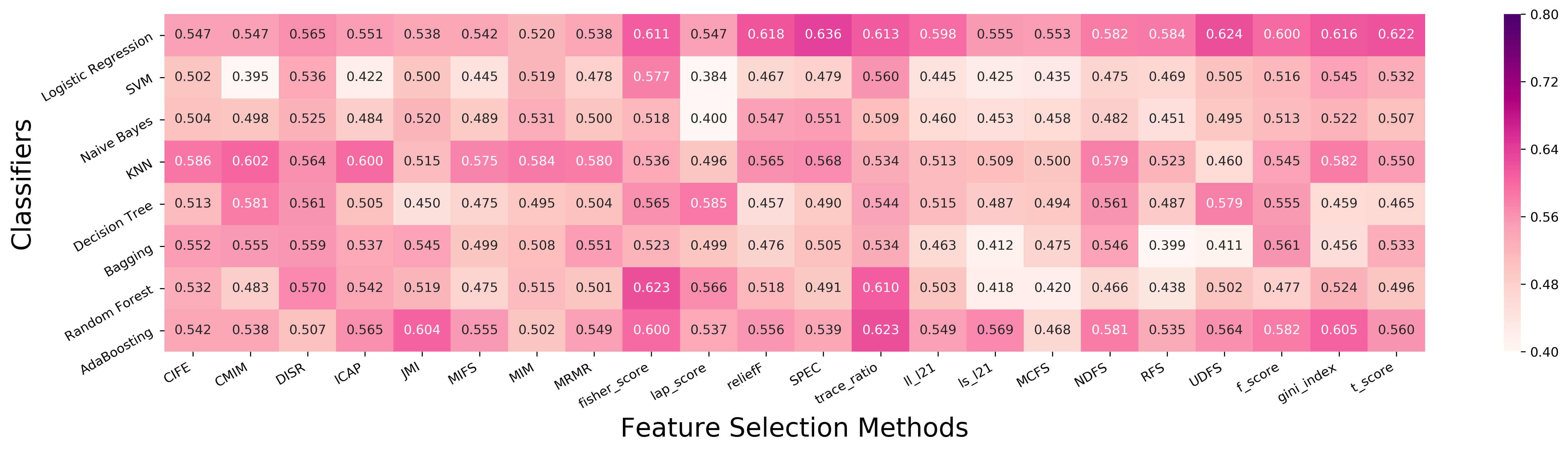


Figure S3. The AUC of 176 models only used top 10 radiomic features.

**Figure S4**


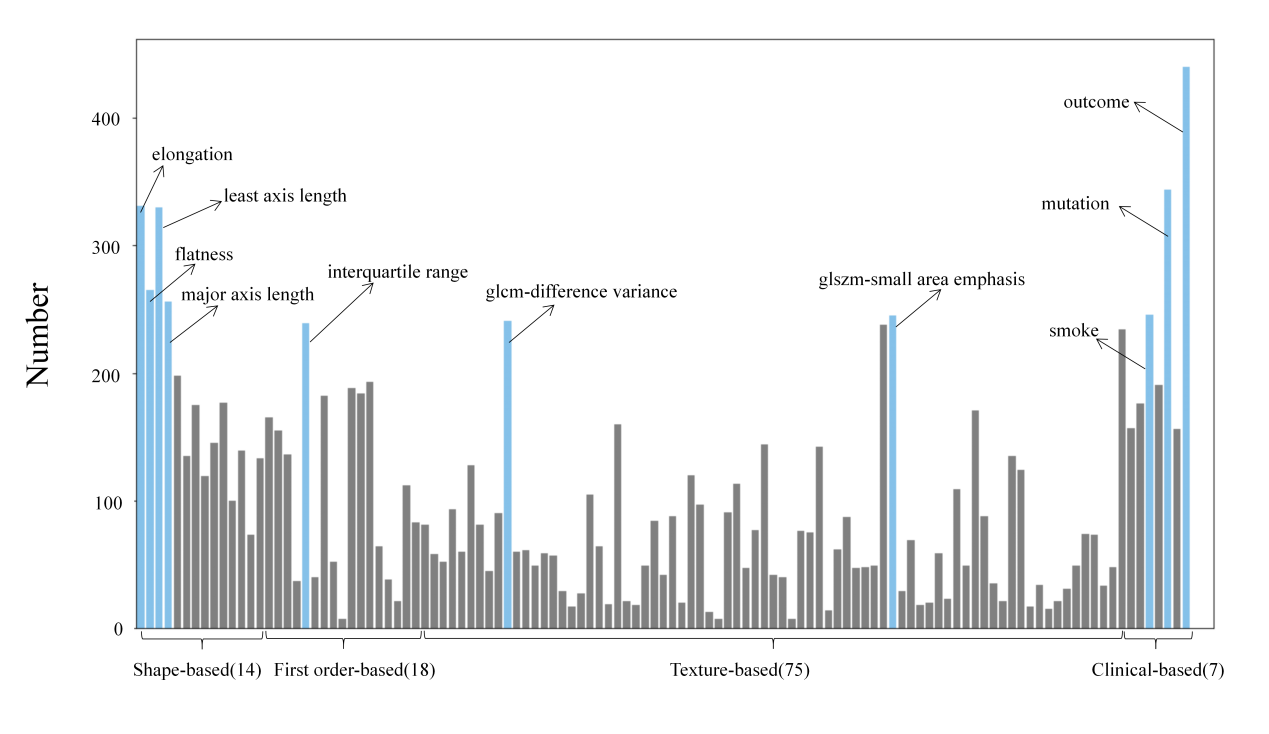


Figure S4. Histogram of the number of times that each feature is selected in the five-fold cross validation. The top-10 features are highlighted as blue.

**References:**

[1].Gavin Brown,A.P.M.Z.and An.A Unifying Framework for Information Theoretic Feature Selection.J MACH LEARN RES. 2012; 13:27-66.

[2].Jundong L,C Kewei and W Suhang.Feature Selection: A Data Perspective.ACM COMPUT SURV. 2017; 50:1-45.

[3].Yao C et al.LLE Score: A New Filter-Based Unsupervised Feature Selection Method Based on Nonlinear Manifold Embedding and Its Application to Image Recognition. IEEE Transactions on Image Processing, 2017. 26(11): p. 5257-5269.

[4].Zheng Z,W Lei and L. Huan.On Similarity Preserving Feature Selection. IEEE Transactions on Knowledge & Data Engineering. 2013; 25:619-632.

[5].Zheng Z and L Huan.Spectral Feature Selection for Supervised and Unsupervised Learning.In:Machine Learning, Twenty-fourth International Conference, 2007.

[6].Feiping N, X Shiming and J Yangqing.Trace Ratio Criterion for Feature Selection.In:National Conference on Artificial Intelligence, 2008.

[7].Jun L,J Shuiwang and Y Jieping.Multi-Task Feature Learning Via Efficient 2,1-Norm Minimization.In:Proceedings of the Twenty-Fifth Conference on Uncertainty in Artificial Intelligence. Montreal, Quebec, Canada: AUAI Press, 2009; 339-348.

[8].Deng C, Z Chiyuan and H Xiaofei.Unsupervised Feature Selection for Multi-Cluster Data.In:Acm Sigkdd International Conference on Knowledge Discovery & Data Mining, 2010.

[9].Li Z and J Tang.Unsupervised Feature Selection via Nonnegative Spectral Analysis and Redundancy Control. IEEE Transactions on Image Processing, 2015. 24(12): p. 5343-5355.

[10]. Feiping N et al. Efficient and Robust Feature Selection via Joint 2,1-Norms Minimization.In:International Conference on Neural Information Processing Systems, 2010.

[11]. Yi Y et al.2,1-Norm Regularized Discriminative Feature Selection for Unsupervised Learning.In:International Joint Conference on Artificial Intelligence, 2011.

[12]. Pedregosa F, Ga, Varoquaux L, et al. Scikit-learn: Machine Learning in Python. J MACH LEARN RES. 2011; 12:2825-2830.
